# The Comparison of Facial Estethics between Orthodontically Treated Patients and Their Parents

**DOI:** 10.1155/2013/903507

**Published:** 2013-10-03

**Authors:** Sertac Aksakalli, Abdullah Demir

**Affiliations:** ^1^Department of Orthodontics, Faculty of Dentistry, Bezmialem Vakif University, Fatih, 34093 Istanbul, Turkey; ^2^Department of Orthodontics, Faculty of Dentistry, Mevlana University, Konya, Turkey

## Abstract

Orthodontists emphasize the importance of facial esthetics while planning a treatment, and orthodontist state that orthodontics have more than expected effects on dentofacial esthetics. The facial esthetics of treated patients and their parents was analyzed and compared to define facial growth and to use in forensic sciences. Our study was applied to 45 orthodontic patients who were treated in our clinic and their untreated parents. The patients were divided into Classes I, II, and III groups according to their malocclusions. Pre- and posttreatment changes, pretreatment facial esthetics of the paitents and its accordance with their parents, and the calculation of heritability tests were performed. After the statistics, for pre- and posttreatment changes, all the groups except Class I revealed significant changes. There were significant correlations of patients for the heritability values and pretreatment esthetic in accordance with parents, but there were more correlations of fathers when compared to mothers. The facial esthetics in adolescences is related with so many factors, not only related with one factor. The facial esthetics in fact includes the aim of evaluation of facial properties partly or totally. Because orthodontic treatments affect facial esthetics, performing similar studies for the treatment outcomes, capabilities, and borders is important.

## 1. Introduction

When someone looks at a baby's face, the question arises: whom does the baby resemble? Particularly with respect to facial morphology, heredity may play an important role in finding an answer [1]. Recently, a more scientific approach combining soft tissue analysis and heritability studies has been used [2].

Human genetics, the heritability of malocclusion, and craniofacial morphology have been subjects of interest for many researchers. These topics have been investigated among different races and among twins and siblings. Studies of twins, families, and populations have concluded that genetics plays an important role in craniofacial structure and growth [3]. Parental data are considered useful in predicting craniofacial characteristics and can give important clues about heritability between parents and their offspring [4–7]. However, little research has been done regarding parent-child heritability. 

Saunders et al. [8] used lateral cephalograms to compare craniofacial dimensions between parents and their offspring. Their study revealed a high level of correlation between first-degree relatives that is compatible with the polygenic theory of inheritance. The study also showed that the use of multiple measurements from both parents could help predict their child's craniofacial morphology. Nakasima et al. [9] performed a similar study. They used cephalograms of Class II and Class III patients and their parents to assess the role of heredity in malocclusions and concluded that there was a high correlation between parents and their children in both groups. Another cephalogram study [10] measured similarities between parents and their offspring and found that the children's craniofacial morphologies were highly correlated with those of their parents. Ichinose et al. [11] researched the similarity of craniofacial morphology between parents and their offspring and stated that there was significant heritability, especially for maxillofacial variables. Additionally, Zekic [12] analyzed cephalograms to evaluate craniofacial similarity between parents and their children and stated that there were high parent-offspring correlations. To assume an orthodontic case to be successful, there must be observable enhancement of facial esthetics. So, determining the development of soft tissue profile before and after treatment is an important step of orthodontics, and researchers have introduced several soft tissue analysis [13–15]. Video records may be advantageous when evaluating dynamics, but research has shown that there is no difference between photographic and video records [16, 17].

There have been limited studies about the heritability of craniofacial morphology and photographic analysis, and there has been no study combining heritability of facial esthetics and photographic analysis. The aims of this study were to determine the effect of heredity on facial esthetics by examining parents and their offspring in Turkish families and to conduct a study combining heritability and photographic analysis. This study will also help in growth prediction and forensic sciences.

## 2. Materials and Methods

 The materials for this study were collected at the Selçuk University, Faculty of Dentistry. The subjects were 45 Turkish children who were treated in the Department of Orthodontics. Inclusion criteria were as follows: no history of orthodontic treatment, no history of craniofacial or dental trauma, no history of maxillofacial or plastic surgery, healthy parents who were blood relatives (no adopted or stepchildren), no usage of glasses, and the presence of frontal and profile extraoral photographs in our archive. After the children were selected, similar extraoral photographs of their parents were taken. The confirmation for the biological relationship between parents and child was done by questionnaire and identification cards that were given by Turkish government. The parents signed informed consent. The ethics committee at the University of Selcuk approved the study (2011.06).

The children were divided into 3 groups according to Angle's classifications. Group I consisted of children with Class I malocclusions and their parents. Children with Class II malocclusions and their parents were placed into Group II, and those with Class III malocclusions and their parents were placed into Group III. The ages of the participants in the groups are shown in Table 1. All children were treated only with fixed orthodontic methods.

**Figure 1:**
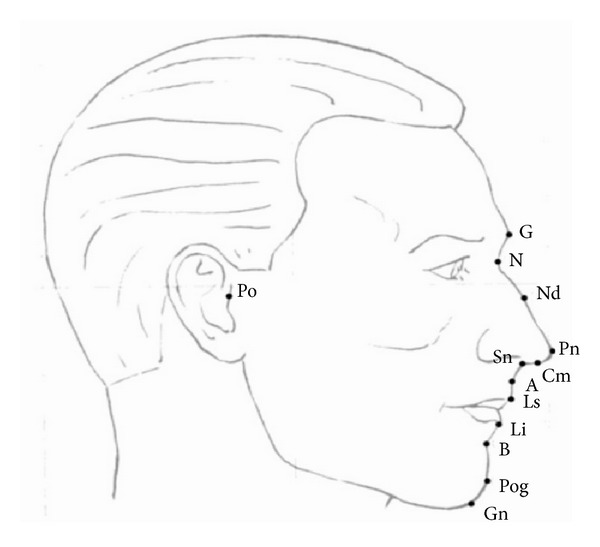
Profile soft tissue landmarks used in this study. G: glabella, N: nasion, Po: porion, Nd: nasal dorsum, Pn: pronasale, Cm: columella, Sn: subnasale, A: A point, Ls: labiale superior, Li: labiale inferior, B: B point, Pog: pogonion, and Gn: gnathion. angles used: nose tip angle (N-Pn-Cm), nasolabial angle (Cm-Sn-Ls), nasomental angle (N-Pn/N-Pog), mentolabial angle (Li-B-Pog), nasofrontal angle (G-N-Nd), total convexity with nose (N-Pn-Pog), total convexity except nose (G-Sn-Pog), soft tissue ANB angle, upper lip projection angle (N-Pog/N-Ls), and upper lip projection angle (N-Pog/N-Li).

**Figure 2:**
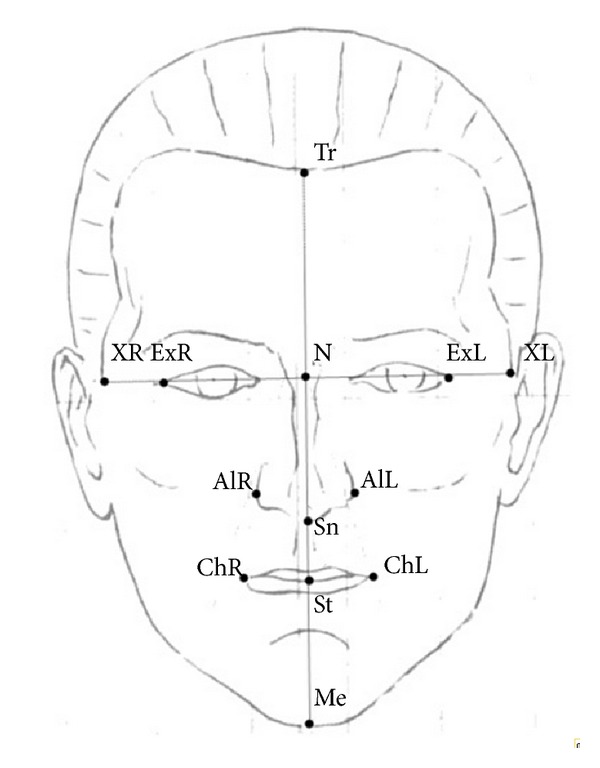
Frontal soft tissue landmarks used in this study. Tr: trichion, N: nasion, Sn: subnasale, ExR: exocanthion right, ExL: exocanthion left, Alr: alare right, AlL: alare left, XR: the most right point according to bipupillary line, and XL: the most left point according to bipupillary line. Ratios used: Tr-N/Sn-Me, N-Sn/Sn-Me, Sn-St/St-Me, XR-XL/Tr-Me, Ex-Me/Ex-Tr, Al-Me/Ex-Al, Al-Me/Ch-Me, Ch-Me/Al-Ch, and ChR-ChL/AlR-AlL.

All photographs were taken with an SLR camera (Nikon D80; Nikon Corporation, Tokyo, Japan) and a telescopic lens (Micro-Nikkor 105 mm; Nikon Corporation). Frontal photographs were taken with the interpupillary plane parallel to the floor, with the teeth in centric occlusion and relaxed facial muscles. Profile photographs were taken with the Frankfort horizontal plane of the soft tissue parallel to the floor and the teeth in centric occlusion. Pretreatment photographs were analyzed using QuickCeph software (QuickCeph Systems, San Diego, CA, USA) by measuring 21 values. Soft tissue landmarks were identified on the profile and frontal aspects of each photograph. These are shown and defined in Figures 1 and 2. All measurements were performed by the same operator (S.A.). For consistency, all measurements were performed by the same operator 1 month later. Method error was assessed by using Dahlberg's method and the coefficient of reliability (Table 2) [18, 19].

After soft tissue values were measured, calculations were performed using 2 statistical methods: the correlation coefficient analysis and the heritability test. Statistical evaluations were performed using the Statistical Package for Social Sciences version 17.0 with the level of significance, *P*, set at 0.05 (SPSS, Chicago, IL, USA). Heritability between parents and their offspring was determined as the value twice the regression coefficient,  *b*, of the offspring on the parent: *h* ^2^ = 2 × *b*  [20]. Heritability estimates must fall between 0 and 1. A heritability estimate of 1 means that the trait is expressed theoretically with no environmental effect; on the other hand, an estimate of 0 defines the trait as having no heritable influence. However, heritability estimates can exceed 1 because in humans, the method used may operate under some simplifying assumptions that can be incorrect or because of sampling fluctuation or environmental variation [7, 9, 21]. 

## 3. Results

### 3.1. Correlation Coefficient between Parents and Offspring

Results of the correlation analysis between parents and their offspring are presented in Table 3 for the Class I group, Table 4 for the Class II group, and Table 5 for the Class III group. For Class I patients, when compared to the Class II and III groups, less significant values were observed. Statistically significant correlations were found more often in the father-offspring group than in the mother-offspring group. Stronger correlations were found for angular measurements related to the nose and upper lip (N-Pn-Cm and Cm-Sn-Ls).

In the Class II group, significant correlations were found more often in the father-offspring group than in the mother-offspring group. The highest correlated values were observed in proportional measurements (Sn-St/St-Me, Al-Me/Ch-Me, and Ch-Me/Al-Ch). Nine measurements were significant in the father-offspring group, while only 2 measurements were significant in the mother-offspring group. 

In the Class III group, significant correlations were found more often in the mother-offspring group than in the father-offspring group. The highest correlated values were observed in 1 proportional and 1 angular measurement (XR-XL/Tr-Me, and A-N-B). Two measurements were significant in the father-offspring group, while 4 measurements were significant in the mother-offspring group. 

### 3.2. Heritability Estimates between Parents and Offspring

When all heritability estimates were summarized, father-offspring *h* ^2^  values were higher than those in the mother-offspring group. In the Class I group, only 1 value was significant in the father-offspring group (N-Pog/N-Ls). There was no significant value in the mother-offspring group (Table 6).

In the Class II group, there was 1 significant value in the father-offspring group (N-Pn-Pog) and 1 in the mother-offspring group (XR-XL/Tr-Me) (Table 7).

In the Class III group, there were 2 significant values in the mother-offspring group (ChR-ChL/AlR-AlL and N-Po-Sn). There were no significant values in the father-offspring group (Table 8).

## 4. Discussion

The primary aim of this study was to examine the resemblance of soft tissue facial esthetics between Turkish parents and their offspring. The ages of the offspring chosen were between 10 and 14. Livson et al. [22] researched changes of craniofacial morphology in parent-offspring groups from birth to adulthood and stated that from age 8 to 18, correlation coefficients between offspring and their parents changed very little. In another study, significant correlation was found between parents and their offspring. It was reported that there were small changes of correlation during growth and that these changes had very little effect on the confidence levels of the subjects in the study groups [23].

In this study, sex differences have not been considered. Pubertal peak stages can be different for boys and girls, and Halazonetis [24] stated that the difference at any pubertal stage was meaningless. However, mother-offspring and father-offspring groups have been analyzed [12, 25].

Anthropometrics, silhouettes, photographs, videos, and cephalograms can be used to evaluate facial esthetics. Using photographs for facial analysis can be logical because they are easier to study than anthropometrics, cover more area than silhouettes, are cheaper than 3-dimensional records, and give no radiation as cephalograms do [16, 26, 27].

According to O'Neill et al. [28] different treatment types were not taken into account. In their questionnaire study, they stated that the type of appliance treatment had no effect on facial esthetics. In another study, Işiksal et al. [29] researched the effect of extraction and nonextraction treatments and concluded that the type of treatment had no effect on smile esthetics.

Facial esthetics results from the combination of both hereditary and environmental effects and is multifactorial. Heritability is obtained from the parent-offspring correlation and shows the ratio of the total phenotype that is contributed by additive genetic variance, the genotype. This additive component is the factor that determines the level of resemblance between relatives, representing the ratio of genetic variance that can be used to predict the expected value in an individual from observations of relatives [30].

Heritability measurements should be between 0 and 1. Heritability of 0 can be reached if no genetic variation is applicable. If no environmental differences are detected in the sample, heritability could approach 1. However, estimates may exceed this range, as we can see in the results of this study, and then are considered meaningless values. This can happen as the result of environmental covariation or sampling fluctuation [31–33] and can be explained by a “cohabitational effect” that causes family members to resemble each other due to both genetic and environmental factors. Sharing the same environment for extended periods can influence phenotypic similarities and enhance phenotypic correlations [34]. This can apply to Turkish families, which often have closer living arrangements than western families do. In our study, when the heritability values were greater than 1, we did not use them for evaluation, as a similar study did [34].

A general evaluation of the correlation results showed a smaller number of statistically significant correlations between parents and their offspring, as well as different *h* ^2^  values for the corresponding measured values. This result is in accordance with the results of a previous study [35]. In general, there were more correlations and higher *h* ^2^  values in the father-offspring groups than in the mother-offspring groups. This result is consistent with the results of other studies [35, 36]. However, there is a study that does not confirm our results which found no significant difference in the value of any parents-offspring correlations [8]. 

Nakata et al. [36] reported that linear measurements had higher heritability estimates than angular measurements did. In our study, proportional (although different from linear) measurements were performed, and angular measurements had higher heritability values. This may be due to variations in sample sizes or age, racial, ethnic, or sex differences. A study [37] investigating heritability values in dizygotic twins found that facial form (XR-XL/Tr-Me) had a strong genetic influence. Our results for the Class II group agree with this.

This study has some limitations. More measurements could be performed, or different races or ethnicities could be selected. The sample size could be increased, but in this kind of studies it was difficult to perform a retrospective study on only a group of patients treated with fixed mechanics. It was also difficult to convince both parents and take photographs of them.

Generally, soft tissues reflect the skeletal unit underlying them, and there were important connections between the amount of hard tissue and the number of changes in the soft tissue [14]. In this paper, we found it valuable to analyze parental data to predict soft tissue growth and heritability. Except predicting facial growth, this kind of studies will also help forensic dentists or researchers to determine facial morphology of a skull according to his/her parents' facial esthetic values.

## 5. Conclusions

In facial esthetics, there are several soft tissue characteristics that are correlated. These characteristics are heritable between parents and their offspring. In this study, significant heritability values were observed for Classes II and III groups. In the Class II group, father-offspring correlations were more common, whereas in the Class III group, mother-offspring correlations were more common. Our findings confirmed that facial soft tissue esthetics is the result of interaction between hereditary and environmental factors.

## Figures and Tables

**Table 1:** Mean ages and treatment times of the groups.

	*N*	Mean pretreatment age	Min	Max	Standard deviation	Total treatment time
Group I	15	12.6	11.1	14	0.66	1.2
Group II	15	11.9	10.9	13.6	0.6	1.9
Group III	15	11.6	11	12.8	0.48	2.3

**Table 2:** Methods errors for measurements used in this study.

Measurements	Dahlberg's method	Coefficient of reliability
Profile photograph analysis		—
Tr-N/Sn-Me (r)	0.02	0.976
N-Sn/Sn-Me (r)	0.01	0.94
Sn-St/St-Me (r)	0.01	0.955
XR-XL/Tr-Me (r)	0.01	0.989
Ex-Me/Ex-Tr (r)	0.02	0.995
Al-Me/Ex-Al (r)	0.07	0.983
Al-Me/Ch-Me (r)	0.02	0.98
Ch-Me/Al-Ch (r)	0.05	0.9
ChR-ChL/AlR-AlL (r)	0.02	0.943
Profile photograph analysis		
N-Pn-Cm (d)	0.37	0.971
Cm-Sn-Ls (d)	0.68	0.983
N-Pn/N-Pog (d)	0.74	0.927
Li-B-Pog (d)	1.58	0.977
G-N-Nd (d)	0.32	0.946
N-Pn-Pog (d)	0.69	0.991
G-Sn-Pog (d)	1.34	0.929
A-N-B (d)	0.31	0.938
N-Pog/N-Ls (d)	0.19	0.988
N-Pog/N-Li (d)	0.33	0.975
N-Po-Sn (d)	0.5	0.979
Sn-Po-Gn (d)	0.64	0.9

d: degree; r: ratio.

**Table 3:** Correlation coefficient values for Group I.

Measurement	Father/offspring	Mother/offspring
	CC	CC
Tr-N/Sn-Me (r)	0.317	0.473
N-Sn/Sn-Me (r)	0.075	0.091
Sn-St/St-Me (r)	0.233	0.094
XR-XL/Tr-Me (r)	0.181	0.014
Ex-Me/Ex-Tr (r)	0.205	0.482
Al-Me/Ex-Al (r)	0.427	0.155
Al-Me/Ch-Me (r)	0.399	0.15
Ch-Me/Al-Ch (r)	0.298	0.206
ChR-ChL/AlR-AlL (r)	0.233	0.472
**N-Pn-Cm (d)**	0.521*	0.528*
**Cm-Sn-Ls (d)**	0.529*	0.072
N-Pn/N-Pog (d)	0.285	0.035
Li-B-Pog (d)	0.44	0.359
G-N-Nd (d)	0.069	0.16
N-Pn-Pog (d)	0.017	0.342
G-Sn-Pog (d)	0.235	0.176
A-N-B (d)	0.339	0.068
N-Pog/N-Ls (d)	0.481	0.034
N-Pog/N-Li (d)	0.178	0.183
N-Po-Sn (d)	0.4	0.46
Sn-Po-Gn (d)	0.364	0.059

CC: correlation coefficient; *P < 0.05; **P < 0.010; ***P < 0.001, r: ratio; d: degree.

**Table 4:** Correlation coefficient values for Group II.

Measurement	Father/offspring	Mother/offspring
	CC	CC
Tr-N/Sn-Me (r)	0.76	0.269
N-Sn/Sn-Me (r)	0.248	0.475
**Sn-St/St-Me (r)**	0.727***	0.306
**XR-XL/Tr-Me (r)**	0.365	0.565*
Ex-Me/Ex-Tr (r)	0.093	0.474
Al-Me/Ex-Al (r)	0.267	0.041
**Al-Me/Ch-Me (r)**	0.764***	0.499*
**Ch-Me/Al-Ch (r)**	0.740***	0.252
ChR-ChL/AlR-AlL (r)	0.19	0.28
N-Pn-Cm (d)	0.007	0.127
Cm-Sn-Ls (d)	0.172	0.369
**N-Pn/N-Pog (d)**	0.71**	0.34
Li-B-Pog (d)	0.111	0.006
**G-N-Nd (d)**	0.606*	0.058
**N-Pn-Pog (d)**	0.624**	0.222
**G-Sn-Pog (d)**	0.765**	0.447
**A-N-B (d)**	0.797**	0.446
**N-Pog/N-Ls (d)**	0.631**	0.197
N-Pog/N-Li (d)	0.458	0.142
N-Po-Sn (d)	0.415	0.431
Sn-Po-Gn (d)	0.132	0.183

CC: correlation coefficient; *P < 0.05; **P < 0.010; ***P < 0.001; r: ratio; d: degree.

**Table 5:** Correlation coefficient values for Group III.

Measurement	Father/offspring	Mother/offspring
	CC	CC
Tr-N/Sn-Me (r)	0.037	0.134
N-Sn/Sn-Me (r)	0.426	0.31
Sn-St/St-Me (r)	0.471	0.248
**XR-XL/Tr-Me (r)**	0.137	0.724***
Ex-Me/Ex-Tr (r)	0.334	0.36
Al-Me/Ex-Al (r)	0.279	0.377
Al-Me/Ch-Me (r)	0.187	0.276
Ch-Me/Al-Ch (r)	0.323	0.105
**ChR-ChL/AlR-AlL (r)**	0.588*	0.288
N-Pn-Cm (d)	0.452	0.087
**Cm-Sn-Ls (d)**	0.58*	0.394
N-Pn/N-Pog (d)	0.283	0.264
Li-B-Pog (d)	0.736	0.289
G-N-Nd (d)	0.36	0.343
N-Pn-Pog (d)	0.304	0.124
G-Sn-Pog (d)	0.192	0.462
**A-N-B (d)**	0.315	0.785**
N-Pog/N-Ls (d)	0.137	0.437
N-Pog/N-Li (d)	0.458	0.18
**N-Po-Sn (d)**	0.012	0.527*
**Sn-Po-Gn (d)**	0.185	0.563*

CC: correlation coefficient; *P < 0.05; **P < 0.010; ***P < 0.001; r: ratio; d: degree.

**Table 6:** Heritability estimates for Group I.

Measurement	Father		Mother	
	*h* ^2^	SE	*h* ^2^	SE
Tr-N/Sn-Me (r)	0.634	0.29	0.958	0.21
N-Sn/Sn-Me (r)	0.18	0.08	0.22	0.33
Sn-St/St-Me (r)	0.44	0.32	0.12	0.35
XR-XL/Tr-Me (r)	0.38	0.28	0.06	0.28
Ex-Me/Ex-Tr (r)	0.14	0.2	0.79	0.28
Al-Me/Ex-Al (r)	0.77	0.2	0.3	0.2
Al-Me/Ch-Me (r)	0.76	0.21	0.2	0.28
Ch-Me/Al-Ch (r)	0.52	0.2	0.3	0.31
ChR-ChL/AlR-AlL (r)	0.26	0.34	0.8	0.22
N-Pn-Cm (d)	0.66	0.22	0.68	0.28
**Cm-Sn-Ls (d)**	1.04^∗∗∗b^	0.19	0.02	0.21
N-Pn/N-Pog (d)	0.59	0.2	0.15	0.21
Li-B-Pog (d)	0.79	0.18	0.5	0.37
G-N-Nd (d)	0.22	0.1	0.3	0.19
N-Pn-Pog (d)	0.69	0.22	0.72	0.21
G-Sn-Pog (d)	0.4	0.28	0.25	0.22
A-N-B (d)	0.75	0.26	0.31	0.31
**N-Pog/N-Ls (d)**	0.83**	0.41	0.21	0.32
N-Pog/N-Li (d)	0.24	0.27	0.26	0.26
N-Po-Sn (d)	0.64	0.24	0.69	0.21
Sn-Po-Gn (d)	0.72	0.22	0.09	0.26

*h* ^2^: heritability estimates; SE: standard error.

^b^Meaningless value; *P < 0.05; **P < 0.010; ***P < 0.001; r: ratio; d: degree.

**Table 7:** Heritability estimates for Group II.

Measurement	Father		Mother	
	*h* ^2^	SE	*h* ^2^	SE
Tr-N/Sn-Me (r)	0.15	0.12	0.538	0.182
N-Sn/Sn-Me (r)	0.88	0.3	0.3	0.29
**Sn-St/St-Me (r)**	1.4^∗b^	0.12	0.42	0.12
**XR-XL/Tr-Me (r)**	0.32	0.18	0.81**	0.21
Ex-Me/Ex-Tr (r)	0.18	0.23	0.79	0.27
Al-Me/Ex-Al (r)	0.54	0.38	0.29	0.6
Al-Me/Ch-Me (r)	1.2	0.12	0.42	0.17
Ch-Me/Al-Ch (r)	1.37	0.1	0.21	0.22
ChR-ChL/AlR-AlL (r)	0.36	0.18	0.54	0.29
N-Pn-Cm (d)	0.04	0.2	0.24	0.28
**Cm-Sn-Ls (d)**	0.84*	0.25	0.46	0.27
N-Pn/N-Pog (d)	1.2	0.17	0.6	0.13
Li-B-Pog (d)	0.09	0.11	0.22	0.3
**G-N-Nd (d)**	1.12^∗b^	0.11	0.09	0.15
**N-Pn-Pog (d)**	0.84**	0.19	0.07	0.19
**G-Sn-Pog (d)**	1.3^∗∗b^	0.16	0.17	0.25
**A-N-B (d)**	1.29^∗∗b^	0.17	0.34	0.19
**N-Pog/N-Ls (d)**	1.24^∗∗b^	0.18	0.56	0.28
N-Pog/N-Li (d)	1.02	0.31	0.48	0.27
N-Po-Sn (d)	0.61	0.2	0.64	0.32
Sn-Po-Gn (d)	0.6	0.27	0.54	0.3

*h* ^2^: heritability estimates; SE: standard error.

^b^Meaningless value; *P < 0.05; **P < 0.010; ***P < 0.001; r: ratio; d: degree.

**Table 8:** Heritability estimates for Group III.

Measurement	Father		Mother	
	*h* ^2^	SE	*h* ^2^	SE
Tr-N/Sn-Me (r)	0.11	0.28	0.974	0.249
N-Sn/Sn-Me (r)	0.14	0.4	0.76	0.3
Sn-St/St-Me (r)	0.77	0.3	0.12	0.22
**XR-XL/Tr-Me (r)**	0.12	0.2	1.3^∗∗b^	0.2
Ex-Me/Ex-Tr (r)	0.66	0.2	0.72	0.26
Al-Me/Ex-Al (r)	0.6	0.33	0.78	0.26
Al-Me/Ch-Me (r)	0.1	0.29	0.49	0.31
Ch-Me/Al-Ch (r)	0.62	0.3	0.06	0.17
**ChR-ChL/AlR-AlL (r)**	1.2^∗∗b^	0.26	0.9**	0.2
N-Pn-Cm (d)	0.91	0.41	0.18	0.4
**Cm-Sn-Ls (d)**	1.01^∗b^	0.21	0.53	0.26
N-Pn/N-Pog (d)	0.52	0.21	0.48	0.32
**Li-B-Pog (d)**	1.33^∗∗b^	0.2	0.52	0.2
G-N-Nd (d)	0.74	0.16	0.72	0.22
N-Pn-Pog (d)	0.59	0.18	0.09	0.29
G-Sn-Pog (d)	0.31	0.23	0.82	0.37
**A-N-B (d)**	0.32	0.24	1.44^∗∗b^	0.34
N-Pog/N-Ls (d)	0.11	0.32	0.77	0.53
N-Pog/N-Li (d)	0.82	0.22	0.09	0.3
**N-Po-Sn (d)**	0.03	0.22	0.82*	0.36
**Sn-Po-Gn (d)**	0.42	0.42	1.06^∗b^	0.29

*h* ^2^: heritability estimates; SE: standard error.

^b^Meaningless value; *P < 0.05; **P < 0.010; ***P < 0.001; r: ratio; d: degree.
